# Perceptual learning in the identification of lung cancer in chest radiographs

**DOI:** 10.1186/s41235-020-0208-x

**Published:** 2020-02-03

**Authors:** Li Z. Sha, Yi Ni Toh, Roger W. Remington, Yuhong V. Jiang

**Affiliations:** 1 Department of Psychology, University of Minnesota, N240 Elliott Hall, 75 East River Road, Minneapolis, MN 55455 USA; 2 School of Psychology, University of Queensland, Brisbane, Australia

## Abstract

Extensive research has shown that practice yields highly specific perceptual learning of simple visual properties such as orientation and contrast. Does this same learning characterize more complex perceptual skills? Here we investigated perceptual learning of complex medical images. Novices underwent training over four sessions to discriminate which of two chest radiographs contained a tumor and to indicate the location of the tumor. In training, one group received six repetitions of 30 normal/abnormal images, the other three repetitions of 60 normal/abnormal images. Groups were then tested on trained and novel images. To assess the nature of perceptual learning, test items were presented in three formats – the full image, the cutout of the tumor, or the background only. Performance improved across training sessions, and notably, the improvement transferred to the classification of novel images. Training with more repetitions on fewer images yielded comparable transfer to training with fewer repetitions on more images. Little transfer to novel images occurred when tested with just the cutout of the cancer region or just the background, but a larger cutout that included both the cancer region and some surrounding regions yielded good transfer. Perceptual learning contributes to the acquisition of expertise in cancer image perception.

## Significance

We explored how people learned to detect tumors on cancer images. Novices classified chest radiographs over four sessions. Unlike perceptual learning of simple visual features, learning of complex features in chest radiographs supported classification of novel images. Transfer to novel images depended on the presentation of both the tumor and some of its surrounding regions; little transfer was observed when tested with just the cutout of the tumor or just the background. These results clarify the nature of perceptual learning of complex radiological images and may provide conceptual underpinning for future innovative technologies that enhance cancer image perception.

## Introduction

The human brain retains remarkable plasticity well into adulthood. Aptly illustrating this plasticity are extensive psychophysical and neuroscience studies on perceptual learning (Fahle, 2005; Gilbert, Sigman, & Crist, 2001; Li, Piëch, & Gilbert, 2004). These studies show that practice on simple stimuli such as Gabor patches or moving dots lead to improved orientation, contrast, or motion sensitivity. Such improvement is often specific to the orientation or location of the stimuli, suggesting that early visual areas such as V1 maintain neural plasticity in adults (Fahle, 2005; but see Xiao et al., 2008). Perceptual learning is not limited to simple stimuli or early visual areas. People can acquire expertise in complex domains, such as in chess and medicine (Charness, Tuffiash, Krampe, Reingold, & Vasyukova, 2005; Cimino, 1999; Ericsson, 2015; Gobet & Simon, 1996). Extensive experience, often over many years, produces domain-specific expertise (Campitelli & Gobet, 2008). Less understood is the nature of perceptual learning of complex stimuli that occurs after several hours of training. This duration is typical of perceptual learning of simple visual features but much too brief to produce expertise in more complex domains. Here we examine whether moderate training on complex images yields specific improvements for just the trained images, or whether training can produce generalizable effects that extend to novel images. Understanding the nature of perceptual learning of complex images may bridge the gap between perceptual learning of simple features and the development of complex expertise. It may also yield practical insights into the training of professionals, such as medical students as they acquire initial perceptual skills in reading radiological images.

The experiments reported here focus on perceptual learning of chest radiographs typical of those used in diagnosing cancerous lung tumors. These images are not only an important class of complex stimuli on which experts have spent long periods of training, but they are also inherently difficult to process. The diversity of medical images (e.g., 2D versus volumetric images; Williams & Drew, 2019) also makes it difficult to establish a uniform training procedure. Some fields, such as the identification of skin cancer, emphasize a checklist of properties based on known signs of disease (Meyer et al., 1996). Other fields emphasize the development of perceptual pattern templates that support the recognition of normal and abnormal images (van der Gijp et al., 2014). Perceptual learning can also complement conceptual knowledge, such as in the identification of melanoma (Xu, Rourke, Robinson, & Tanaka, 2016).

Perceptual learning may be particularly important for accurate reading of radiological images. On radiographic images, lung cancer or breast cancer often presents as a slight change from the appearance of normal tissues and is embedded in a much larger image of the lung or the breast. Recognition of cancer on chest radiographs or mammograms entails visual search and perceptual segmentation (Drew, Evans, Võ, Jacobson, & Wolfe, 2013; Krupinski, 2010). Search consists of two interactive processes (Drew et al., 2013; Kundel, Nodine, Conant, & Weinstein, 2007). The first is global image analysis, which extracts overall characteristics of the image, much as the analysis of the gist of a natural scene. This is followed by detailed image scanning involving a sequence of fixations and attentional shifts (Wolfe, Võ, Evans, & Greene, 2011). Evidence suggests that global image analysis can support above-chance classification of mammograms. Expert mammographers were shown to be able to classify an image as normal or abnormal after viewing it for just 250 ms, even though the experts could not reliably localize the tumor (Drew et al., 2013; Evans, Haygood, Cooper, Culpan, & Wolfe, 2016; Nodine et al., 1999). Experts can also classify a breast image as abnormal even when the lesion region is removed, or when viewing a normal breast contralateral to the cancerous one (Evans et al., 2016). These findings suggest that surrounding regions contain signals that are correlated with the presence of tumors. Apparently, both initial global analysis and subsequent search and segmentation phases of visual search contribute to the detection of cancerous tumors.

Two studies demonstrate that perceptual training enhances cancer detection on radiological images. Sowden, Davies, and Roling (2000) trained participants three times a day, over 4 days, using 60 mammograms. Half of the participants viewed the images in positive contrast and the other half viewed the images in negative contrast. They clicked on the region that contained a microcalcification cluster and received feedback on their localization accuracy. Performance improved over the four training days. On the 5th day, participants viewed the same 60 mammograms, but this time in the opposite contrast to that used in training. Performance on the 5th day was better than on the 1st day, but worse than on the 4th day, suggesting that learning was partially retained when the images reversed polarity. It is unclear whether perceptual learning was specific to the trained images, because this study did not test novel images.

Transfer to novel images was demonstrated in a second study of pelvic radiographs. This study trained novices to identify bone fractures on pelvis radiographs (Chen, HolcDorf, McCusker, Gaillard, & Howe, 2017). Participants viewed one image at a time and made a fracture “present/absent” response. Following an incorrect response, the computer marked out the location of the fracture. A series of six experiments varied the number of training images and difficulty. Increasing the number of images used in training yielded greater transfer to novel images, suggesting that high variability in the training set facilitates learning (Mettler & Kellman, 2014). Two factors, however, limit the generalizability of this conclusion to perceptual learning of other radiological images. First, in Chen et al. (2017), novices performed at above-chance levels before receiving training. This suggests that bone fractures may be relatively easy to discern. Second, after just several hundred training trials, the top five novices achieved ~ 90% accuracy, a level comparable to that of board-certified radiologists. In contrast, other radiological tasks require several years of training to achieve the level of an expert (Krupinski, 2010; Myles-Worsley, Johnston, & Simons, 1988; Nodine et al., 1999). Thus, it is unclear whether the effectiveness of perceptual learning observed with bone fracture images also generalizes to other, more difficult diagnostic images.

The current study elucidates the nature of perceptual learning using chest radiographs that may contain evidence of lung cancer. The target signal - a cancerous tumor in the lung - is characterized as a mass rather than calcification. We chose radiographs of the lung because of their clinical significance and because the stimuli are difficult for untrained observers to discern. In fact, before training, participants in our study were at the level of chance in classifying an image as normal or abnormal. Even after 4 days of training, their performance was far below the ceiling level. The use of complex radiological images allowed us to examine the specificity of perceptual learning in a difficult task. This study bridges the gap between perceptual learning of simple features that arises after a few sessions, and complex expertise that requires several years of training.

The three aims of this study support the science of perceptual learning by examining the scope of learning with respect to complexity, as well as the underlying nature and constraints of the learning. First, we tested whether perceptual learning of chest radiographs could induce transferrable effects to novel images. Because exposure to both normal and abnormal images is important for developing pattern recognition (van der Gijp et al., 2014), we trained participants with pairs of images (see also Sunday, Donnelly, & Gauthier, 2017), a normal and an abnormal image, and asked participants to select the abnormal image and localize the tumor. To direct participants’ attention to the tumor properties, we provided feedback on which image was abnormal and where the tumor was. Training participants by forcing them to choose which of two images contains cancer is not representative of how radiologists diagnose images in the clinic. However, the task facilitated perceptual comparison (Krupinski, 2010) and its use was restricted to the training phase.

To examine whether training produced transferrable effects, we administered testing sessions that included both trained and untrained images. Images were presented one at a time in the testing sessions, requiring the participants to report whether the image was normal or abnormal. This task was more similar to actual radiological tasks and served to assess training effectiveness. Relative to the pre-training baseline, improvements on untrained images following training would suggest that training has yielded transferrable effects.

The second goal of this study was to examine the roles of image repetition and image diversity. More repetitions of the same images in training could bootstrap learning by repeating critical image statistics that characterize cancer (Werker & Yeung, 2005). If these statistics are shared with untrained images, bootstrapping based on repeatedly learning the same images will facilitate cancer detection on untrained images. On the other hand, increasing the number of different training images may enhance learning by fine-tuning discrimination to features corresponding to the variability in normal and abnormal tissues. Consistent with this possibility, Chen et al.’s (2017) study on bone fractures found that performance improved with an increasing number of different training images. To evaluate if this pattern also applies to the learning of chest radiographs, we administered two training schedules in separate groups of participants. The 30-image group was trained with 30 normal and 30 cancerous images, whereas the 60-image group was trained with 60 normal and 60 cancerous images. The total number of training trials was the same for the two groups, allowing us to test how image repetition and image variability influence perceptual learning.

The third goal of this study was to investigate whether learning was part-based or holistic. To this end, participants were tested with the full image, a cutout containing only the tumor, or the background after the tumor region has been cut out. Part-based tests are often used to probe whether perception is holistic (Tanaka & Simonyi, 2016). In face perception, for example, participants are worse at judging whether two noses are identical if the noses are displayed in isolation rather than in the context of a face. In contrast, house recognition is part-based (Tanaka & Farah, 1993). Several studies on medical image perception suggest that cancer detection depends, in part, on the global image statistics. As noted earlier, radiologists can rapidly extract the global image statistics to render an initial decision on whether a mammogram may be cancerous (Evans et al., 2016; Evans, Georgian-Smith, Tambouret, Birdwell, & Wolfe, 2013; Nodine et al., 1999). Additionally, presenting mammograms in an inverted orientation impairs radiologists’ performance, suggesting that breast cancer detection is holistic (Chin, Evans, Wolfe, Bowen, & Tanaka, 2018). If perceptual learning of chest radiographs also involves the learning of global image statistics, then performance should decline when just the tumor cutout or just the background is presented.

Alternatively, lung cancer detection may rely on identifying local tumor properties. Local properties may take two forms. First, the tumor itself may contain characteristic properties that distinguish it from normal tissue. If this is the case, then participants should be able to discriminate tumor cutouts from normal cutouts. Second, the local contrast between the tumor and its immediately surrounding region may be a key signal for lung cancer detection. Radiologists rely on a comparison between the tumor and other regions to detect changes that may signal the presence of a tumor (Carmody, Nodine, & Kundel, 1981). If the local contrast is important, then a larger cutout that encompasses both the tumor and some surrounding tissue will be needed for cancer detection.

We conducted three experiments to address these goals. In experiment 1, we trained participants across four consecutive days using either 30 or 60 normal/cancerous images. Testing occurred after each training day and included both trained and untrained images, in one of three formats (full image, tumor only, or background only). This experiment aimed to establish an effective training procedure, clarify the roles of image repetition/variability, and provide initial findings on the local/global nature of learning. Experiment 2 further elucidated the nature of learning by including a larger cutout that encompassed the tumor and immediately surrounding region. Experiment 3 was a replication of the key findings with a larger sample. Together, these experiments provide valuable information on how perceptual learning could be incorporated into efficient training schedules.

## Method

### Participants

A total of 60 healthy adults participated in three experiments. There were 14 men and 46 women with a mean age of 20.6 years (range 18–32 years). All participants had normal or corrected-to-normal visual acuity and were naïve to the purpose of the study. Participants signed an informed consent form and were compensated for their time.

Sample sizes were predetermined (12 participants in each of experiments 1A, 1B, and 2, and 24 participants in experiment 3) to be comparable to typical sample sizes in previous perceptual learning studies.^1^ To further increase statistical power, where justifiable, data from different experiments involving the same conditions were pooled. Combining data across experiments is warranted because participants were tested in the same conditions, and there were no direct comparisons across experiments. Table 1 lists the sample size and the training and testing schedules and formats for each experiment.

**Table 1:** An overview of conditions administered in experiments 1–3

Experiment	Number	Training (4 sessions)	Testing format	Testing sessions
1A	12	30-image × 6 reps/sess.	Intact, small cutout, background	Pretest, 4 post-tests
1B	12	60-image × 3 reps/sess.	Intact, small cutout, background	Pretest, 4 post-tests
2	12	60-image × 3 reps/sess.	Intact, small cutout, large cutout	Pretest, 4 post-tests
3	24	30-image × 6 reps/sess.	Intact, small cutout, background	Pretest, 1 final post-test

*reps/sess.* repetitions per session

### Materials and stimuli

Participants were tested individually in a room with normal interior lighting. The stimuli were presented on a 21″ iMac monitor (1920 × 1080 pixels) using MATLAB and Psychtoolbox (Kleiner, Brainard, & Pelli, 2007). The unconstrained viewing distance was approximately 40 cm.

Chest radiographs were obtained from the Japanese Society of Radiological Technology Database (Shiraishi et al., 2000), accessible at http://db.jsrt.or.jp/eng.php. The entire database contains 154 cancerous images and 93 normal images. The database provides the following information about each cancerous image: the location of the tumor, tumor size, and tumor subtlety on a scale from 1 (most subtle) to 5 (most obvious). Each image is originally 2048 × 2048 pixels in size. In the original images, tumors range from 6 to 60 pixels (1.05–10.5 mm) in diameter, with a mean of 18.4 pixels (3.2 mm). Some images contained a patient label in the upper left or upper right region that we edited out.

In our study, we included 80 cancerous images with a single tumor and 80 normal images without any tumors. The images were adapted to 614 × 614 pixels (training sessions) and 768 × 768 pixels (testing sessions) in size.^2^ All images underwent the same editing process to eliminate patient labels.

To create tumor cutouts used in the testing sessions, we cut out a square region based on the location and size of the tumor provided by the database. The small cutout was a square region with a side length equal to the diameter of the tumor on that image. Because the tumors varied in size, the cutout also varied in size. The remaining image constituted the “background.” The larger cutout that included both the tumor and the immediately surrounding region was a square region with a side length of twice the diameter of tumor on the image. To ensure that the cancerous and normal images had comparable cutouts in terms of location and size, for each cancerous cutout, a square region of the same size and in the same location was cut out from a normal image. The cutouts were only used in the testing sessions, in which one image was presented at a time.

### Experiment 1: training sessions

Participants were trained on four sessions across four consecutive days. Participants were randomly assigned to receive one of the two training schedules. In the 30-image training group (experiment 1A), stimuli for training contained 30 cancerous and 30 normal images randomly selected from the database. In the 60-image training group (experiment 1B), training was done with 60 cancerous and 60 normal images randomly selected from the database. Attempts were made to counterbalance images used in training and testing across participants. For example, if images 1–30 were in the training and images 31–60 were in the testing sessions for one participant, then a second participant would undergo training with images 31–60 and testing with images 1–30. The images were selected such that the distribution of tumor subtlety among images used in the training and testing sessions were representative of the tumor subtlety distribution of the entire image set.

**Fig. 1:**
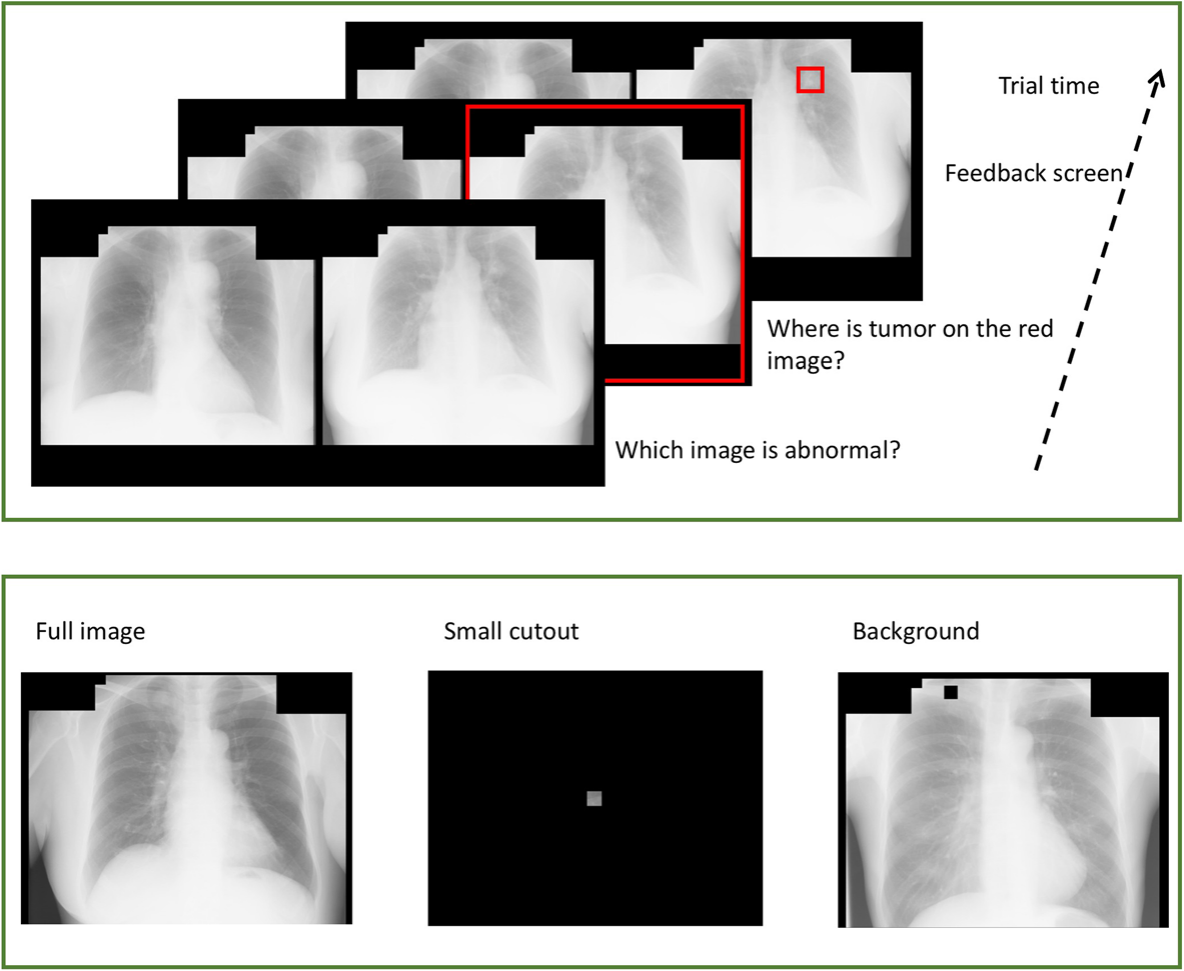
Sample stimuli used in this study. Images were reproduced from the Japanese Society of Radiological Technology Database (Shiraishi et al., 2000). Top: in the training sessions, a pair of normal and abnormal images were presented. Participants clicked on the image that was abnormal. Following their response, a red frame indicated which image was abnormal. Participants then clicked on where they thought the tumor was. This was followed by a feedback screen that indicated the location of the tumor. Bottom: three formats used in the testing sessions

At the beginning of each training session, we randomly paired a cancerous and a normal image for use in a specific trial. On each trial, cancerous and normal images were presented side by side, with the left-right position counterbalanced across trials. Participants first classified the images by clicking on the one that they thought contained a tumor. Upon their response, a red frame highlighted the actual tumor image to provide feedback. Participants then localized the tumor by clicking on where they thought the tumor was on the cancerous image. Once they responded, a small red outline frame of the same size as the tumor highlighted the actual tumor location. Each pair of images was presented three times in the 60-image training group, and six times in the 30*-*image training group, giving a total of 180 trials per training session. The pairing was consistent within a training session. The images were re-paired at the beginning of each session, meaning that the pairing changed on subsequent days. Figure 1 (Top) illustrates the training procedure.

### Experiment 1: testing sessions

**Fig. 2:**
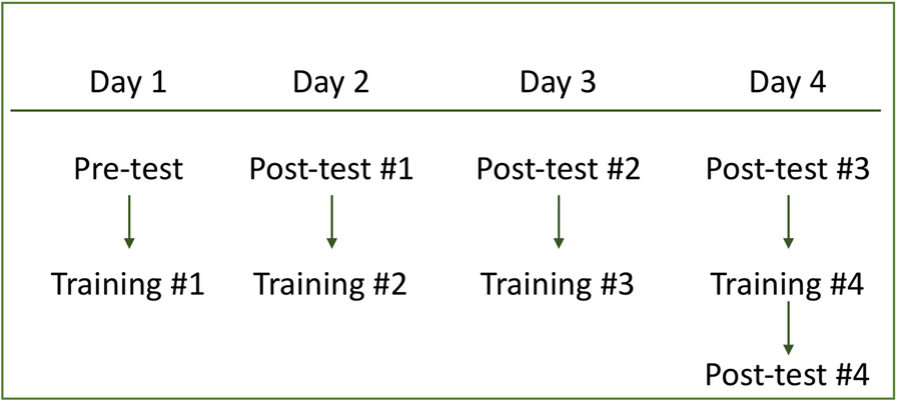
The training and testing schedule used in experiments 1A, 1B, and 2. Participants in experiment 3 completed only two of the five testing sessions - a pre-test on day 1 and a final post-test on day 4

A pre-test and four post-test sessions were administered (Fig. 2). In each testing session, participants viewed one image at a time and pressed a button to indicate whether it came from a patient with cancer (“y” key) or not (“n” key). They did not receive any feedback about the accuracy of the response.

Each testing session comprised a factorial design of three factors, namely training history (trained versus untrained images), disease status (normal versus abnormal images), and image format (intact, cutout, or background). The inclusion of three formats allowed us to examine whether cancer detection depended on local tumor properties or signals that extend beyond the tumor region. The test image could be displayed in its entirety (intact), or it may include just the region containing the tumor (cutout), or the remaining region (background).

There was a total of five testing sessions: a pre-test before the day-1 training, and four post-tests, one after each day of training (Fig. 2). Each testing session contained 180 trials, and was based on 15 normal and 15 abnormal images randomly drawn from the trained set and 15 normal and 15 abnormal images randomly drawn from the untrained set. Because the image database contained a total of 80 normal and 80 abnormal images, some untrained images were presented more than once across the five testing sessions. On average, participants in experiment 1A (30-image training schedule) saw a specific untrained image 1.5 times in testing, and participants in experiment 1B (60-image training schedule) saw a specific untrained image 3.75 times in testing.

### Experiment 2

This experiment was a partial replication of experiment 1B using the same 60-image training schedule. In the testing phase, we replaced the background-only format with a larger cutout. Specifically, to retain more of the contrast between the tumor and the background, the test image could be presented in full, or it may be a small cutout of the region containing the tumor, or a large cutout of the tumor including a region four times as large as the tumor (i.e., the side length of the cutout was twice the diameter of the tumor).

### Experiment 3

This experiment was a partial replication of experiment 1A using the same 30-image training schedule*.* We made two changes. First, the sample size in experiment 3 was twice as large as in experiment 1A. Second, to eliminate the repetition of untrained images in the testing sessions, in experiment 3 the number of testing sessions was reduced to two - a pre-test immediately before day-1 training and a final post-test immediately after day-4 training. Each testing session included 25 normal and 25 abnormal images from the trained set and 25 normal and 25 abnormal images from the untrained set. Each image was displayed in one of three formats (full, small cutout, or background), giving a total of 300 trials per testing session. Pre-test and post-test were based on entirely different untrained images. Table 1 summarizes the training schedule and testing conditions across experiments.

## Results

### Training

**Fig. 3:**
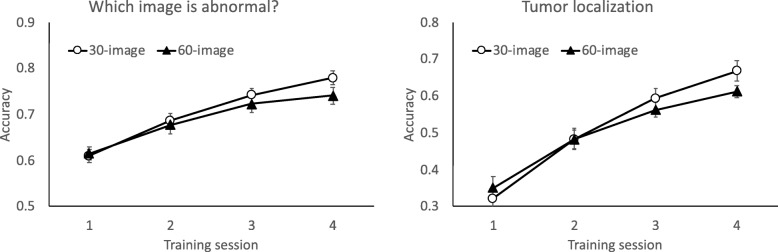
Accuracy across four sessions of training. Left: image classification (which image is abnormal?). Right: tumor localization. Data from the 30-image training schedule came from 36 participants (12 from experiment 1A and 24 from experiment 3). Data from the 60-image training schedule came from 24 participants (12 each in experiments 1B and 2). Error bars show +/− 1 S.E. of the mean

We first examined how performance changed across the four training sessions. Of the 60 participants, 36 (experiments 1A and 3) followed the 30-image training schedule, and 24 (experiments 1B and 2) followed the 60-image training schedule. As shown in Fig. 3, both the 30-image and 60-image training groups improved with training. In the image classification task (which image is abnormal?), analysis of variance (ANOVA) of the training condition (30-image versus 60-image) and training session (1–4) revealed a significant main effect of session, showing improvement across training sessions, *F*(3, 174) = 108.89, *p* < .001, *η*_*p*_^2^ = .65. There was no effect of training schedule, *F* < 1, and the interaction between training schedule and session was not significant, *F*(3, 174) = 2.26, *p* = .084, *η*_*p*_^2^ = .037. In the tumor localization task, performance also improved across training sessions, *F*(3, 174) = 269.34, *p* < .001, *η*_*p*_^2^ = .82 for the main effect of session. The main effect of training schedule was not significant, *F* < 1, qualified by an interaction between session and training condition, *F*(3, 174) = 5.30, *p* < .002, *η*_*p*_^2^ = .084. Thus, both groups improved with training, with evidence of greater improvement in the 30-image group. Although this latter finding was intuitive, results could have differed if the images were not learnable, or if learning had saturated after a small number of repetitions.

Although all chest radiographs had similar anatomic structures, the images contained idiosyncratic properties, such as a slightly darker rib cage on one image or an obvious asymmetry between the left and the right sides on another. Did participants simply remember incidental properties of specific images, or did they learn to recognize tumors on the trained images? To address this question, we examined training performance for tumor images of different subtlety levels. The database gave a rating of 1 to images with the most subtle tumor, up to a rating of 5 to images with the most obvious tumor. If participants simply remembered incidental properties associated with a specific normal or abnormal image, performance should be insensitive to the subtlety of the tumor. But if they learned to recognize a tumor on the trained images, then performance should be higher for images containing a more obvious tumor. On average, the proportion of images having subtlety ratings of 1 through 5 was 7.50%, 13.75%, 37.50%, 32.50%, and 8.75%, respectively. Given the small number of stimuli per subtlety level, this analysis combined data from all 60 participants.

**Fig. 4:**
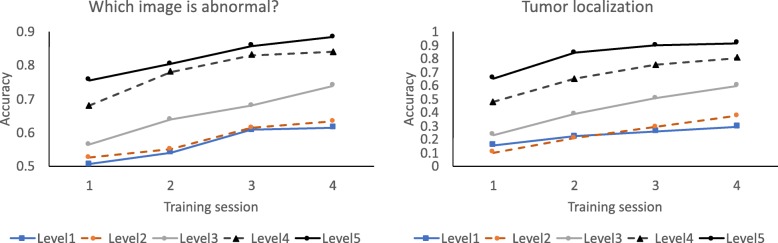
Training performance for displays containing tumors of different subtlety levels. Level 1 is the most subtle, and level 5 is the most obvious. Data were the average of all 60 participants

As shown in Fig. 4, training performance was a monotonic function of tumor subtlety. Image classification was better in later training sessions, *F*(3, 177) = 69.38, *p* < .001, *η*_*p*_^2^ = .54, and for images containing more obvious tumors, *F*(4, 236) = 151.66, *p* < .001, *η*_*p*_^2^ = .72. The two factors did not interact, *F*(12, 708) = 1.32, *p* = .20. Similarly, tumor localization was better in later sessions, *F*(3, 177) = 223.06, *p* < .001, *η*_*p*_^2^ = .79, and for images containing more obvious tumors, *F*(4, 236) = 407.92, *p* < .001, *η*_*p*_^2^ = .87. The two factors interacted, *F*(12, 708) = 13.82, *p* < .001, *η*_*p*_^2^ = .19, with greater training effects for images with intermediate subtlety levels than for images at the extreme ends. This could be due to a ceiling or floor effect. That performance systematically varied across tumor subtlety levels indicates that participants learned to detect tumors on the trained images.

### Testing

The inclusion of untrained images and parts of an image in the testing sessions allowed us to investigate the transfer of learning. The experiments tested several image formats, including a small cutout, a larger cutout, or the background regions without the tumor (Table 1). In the following report, we first examined the transfer to untrained images presented in an intact format. A second analysis focused on transfer to parts of an image. Because the testing sessions used a yes/no tumor classification task, we calculated *d’* as an index of sensitivity (Macmillan & Creelman, 2005). The Appendix contains information about response criterion.

#### Testing with images in an intact format

All participants were tested with trained and untrained images in an intact format (Table 2). Across all participants, *d’* did not differ significantly from 0 in the pre-test session, *t*(59) = 0.24, *p* = .82. Thus, unlike bone fracture (Chen et al., 2017), lung cancer could not be identified reliably before training.

**Table 2:** Mean *d’* for intact images used in the testing sessions

Experiment and training schedule	Testing image	Pre-test	Post-test 1	Post-test 2	Post-test 3	Final test	Pre-test vs. final test
1A (*N* = 12) 30-image	Trained	−0.04	0.67	0.64	0.95	1.58	*P* < .001
	Untrained	−0.10	0.40	0.22	0.68	0.88	*P* < .002
1B (*N* = 12) 60-image	Trained	0.30	0.76	0.96	0.86	0.88	*P* < .001
	Untrained	−0.03	0.47	0.88	0.48	1.01	*P* < .007
2 (*N* = 12) 60-image	Trained	0.18	0.94	0.49	1.01	0.68	*P =* .16^a^
	Untrained	−0.15	0.80	0.54	0.70	0.66	*P* < .005
3 (*N* = 24) 30-image	Trained	−0.11	Not applicable			1.14	*P* < .001
	Untrained	0.08	Not applicable			0.74	*P* < .001

*P* values show results from *t* tests comparing pre-test with the final post-test

^a^In experiment 2, although the pre-test did not differ significantly from the final post-test, it was significantly worse than the average of all four post-tests, *p* < .05

Table 2 shows the mean *d’* in each testing session of each experiment and *p values* comparing the pre-test with the final post-test. Owing to the relatively small number of trials per condition, there was considerable variability in the *d’* data. Nonetheless, in all cases, *d’* improved from the pre-test to post-test for both trained and untrained images. Notably, this finding was observed even when the untrained images occurred only once, as in experiment 3. These data provide compelling evidence that perceptual learning of chest radiographs depicting lung cancer transfers to untrained images.

**Fig. 5:**
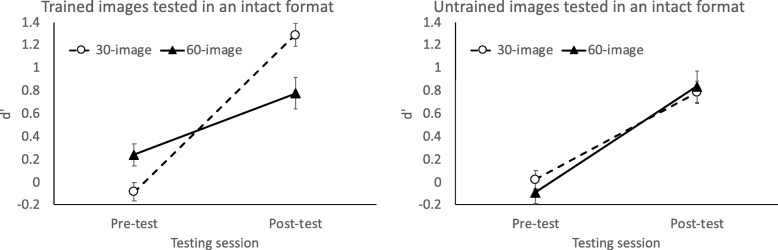
Value of *d’* across pre-test and the final post-test, when tested with intact trained images (left) and intact untrained images (right). Error bars show +/− 1 S.E. of the mean

How did training schedule - 30-image versus 60-image - influence the degree of image-specific versus generalizable learning? Figure 5 (left) shows that when tested with intact trained images, participants in the 30-image group (*N* = 36) improved more than participants in the 60-image group (*N* = 24). The main effect of testing session was significant, *F*(1, 58) = 69.03, *p* < .001, *η*_*p*_^2^ = .54, qualified by a significant interaction between group and testing session, *F*(1, 58) = 13.23, *p* < .001, *η*_*p*_^2^ = .19. In contrast, when tested with intact untrained images (Fig. 5, right), the two groups had comparable gains. Performance improved from pre-test to the final post-test, *F*(1, 58) = 58.11, *p* < .001, *η*_*p*_^2^ = .50, and this effect did not interact with group, *F* < 1. Thus, training with fewer images but greater repetition yielded better learning of the trained images, but the degree of transfer to untrained images was comparable between the 30-image and 60-image groups.

Although participants showed clear evidence of transfer to untrained images as a whole, we found no evidence of generalized learning for the most subtle tumor. At the final post-test, the hit rate for untrained tumor images across all 60 participants was 41%, 49%, 54%, 60%, and 66%, for tumors ranging from the most subtle to the most obvious. The false alarm rate on normal images was 41%. Pairwise comparisons showed significantly more hits than false alarms for tumors at levels 2–5 (*p* < .005 for all), but the hit rate for the most subtle tumor was no higher than the false alarm rate (*p* = .92). Four days of training was insufficient to yield significant learning of the most subtle tumors, revealing a potential limitation of the current training approach.

### Testing with tumor cutouts

Did participants learn local properties associated with tumors, or did they rely on global image information? Here we examined classification accuracy for tumor cutouts and the background. All 60 participants were tested with a small cutout that was a square region encompassing the tumor. As was the case with intact images, pre-test performance on small cutouts did not differ from chance, *t*(59) = 0.87, *p* = .39.

**Fig. 6:**
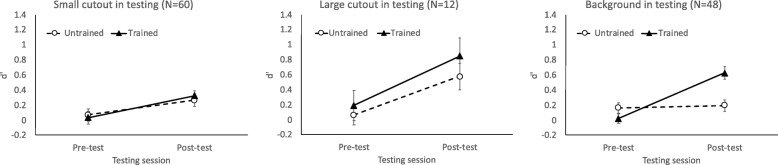
Value of *d’* from the testing sessions involving parts of an image. Left: the small cutout with a side length equal to the tumor diameter. Middle: the large cutout with a side length twice the tumor diameter. Right: the background region after the tumor was cut out. Error bars show +/− 1 S.E. of the mean

As shown in Table 3, improvements from pre-test to the final post-test were inconsistent across experiments, with few statistically significant effects. When data were pooled across all 60 participants, we did observe a significant improvement (Fig. 6, left). ANOVA of image novelty (trained or untrained) and testing session (pre-test or the final post-test) revealed just a significant main effect of session, *F*(1, 59) = 10.93, *p* < .002, *η*_*p*_^2^ = .16, and no effect of image novelty, *F* < 1, or their interaction, *F* < 1.

**Table 3:** Mean *d’* for the small tumor cutout used in the testing sessions

Experiment and training schedule	Testing image	Pre-test	Post-test 1	Post-test 2	Post-test 3	Final test	Pre-test vs. final test
1A (*N* = 12) 30-image	Trained	−0.21	0.34	0.07	0.38	0.27	*P* = .11
	Untrained	0.00	0.40	0.36	0.15	0.07	*P =* .83
1B (*N* = 12) 60-image	Trained	0.10	0.53	0.47	0.66	0.44	*P =* .13
	Untrained	0.36	0.34	0.33	0.52	0.65	*P* = .38
2 (*N* = 12) 60-image	Trained	0.37	0.39	−0.02	0.43	−0.06	*P =* .04
	Untrained	0.10	0.14	0.53	0.17	0.45	*P* = .17
3 (*N* = 24) 30-image	Trained	−0.04	Not applicable			0.48	*P* < .001
	Untrained	−0.05	Not applicable			0.08	*P* = .30

*P* values show results from *t* tests that compared pre-test with the final post-test

What about the larger tumor cutout encompassing a region four times as large as the tumor itself? This stimulus, tested in experiment 2, contains contrast information between the tumor and the surrounding regions but does not contain additional background information. The 12 participants tested with a large tumor cutout evidenced improvements for both trained and untrained images. As seen in Fig. 6 (middle), *d’* improved from pre-test to the final post-test, *F*(1, 11) = 7.45, *p* = .02, *η*_*p*_^2^ = .40 for the main effect of session. The main effect of image novelty was not significant, *F*(1, 11) = 1.82, *p* = .20, neither did image novelty interact with session, *F* < 1. These data suggest that the small cutout weakly supported tumor detection, whereas the larger cutout enabled tumor detection for both trained and untrained images.

### Testing with the background only

Some of the images used in the testing sessions were the background that had the tumor removed. Here we tested whether an image with the tumor area cutout (“background”) can still support tumor detection.

As shown in Table 4 and Fig. 6 (right), performance improved on trained images but not on untrained images. When data were aggregated across all 48 participants, ANOVA of image novelty (trained or untrained) and testing session (pre-test or final post-test) showed main effects of image novelty, *F*(1, 47) = 5.92, *p* = .02, *η*_*p*_^2^ = .11, and session, *F*(1, 47) = 17.69, *p* < .001, *η*_*p*_^2^ = .27, qualified by a clear interaction, *F*(1, 47) = 19.64, *p* < .001, *η*_*p*_^2^ = .30. Follow-up tests showed that performance improved for trained images, *t*(47) = 6.13, *p* < .001, but not for untrained images, *t*(47) = 0.33, *p* = .75. Thus, learning of the background was specific to the trained images.

**Table 4:** Mean d’ for classifying images with the tumor region removed (“background”)

Experiment and train schedule	Testing image	Pre-test	Post-test 1	Post-test 2	Post-test 3	Final test	Pre-test vs. final test
1A (*N* = 12) 30-image	Trained	0.12	0.79	0.70	0.83	0.87	*P* < .002
	Untrained	0.32	0.37	0.35	0.48	0.33	*P =* .98
1B (*N* = 12) 60-image	Trained	0.18	0.24	0.26	0.73	0.73	*P <* .03
	Untrained	0.26	0.36	0.58	0.30	0.16	*P* = .63
3 (*N* = 24) 30-image	Trained	−0.10	Not applicable			0.44	*P* < .001
	Untrained	0.03	Not applicable			0.14	*P* = .29

*P* values show results from *t* tests that compared pre-test with the final post-test. The overall *d’* during pre-test was not different from chance, *p* = .10

## Discussion

This study investigated the specificity of perceptual learning of chest radiographs. The novices tested in this study performed at chance level before training. This differed from previous studies in which participants were trained to identify bone fracture or skin melanoma (Chen et al., 2017; Xu et al., 2016). The pre-training performance in those studies was above chance, suggesting that the abnormalities were discernible even without training. In our study, novices were unable to identify cancerous images before training, but their performance improved with training. The improvement over four days differed from a previous study in which participants were trained to identify radiographic images (Sowden et al., 2000). In that study, most improvements occurred in the first 2 days of training, with no additional change on days 3 and 4. Three main findings were observed in the present study.

First, our study showed that training yielded generalizable effects. When tested with untrained images presented in an intact format, participants showed clear improvements from the pre-test to the final post-test. As shown in experiment 3, these improvements occurred even when untrained images were not repeated, ruling out any learning of untrained images through repetition in testing. These data provide compelling evidence that perceptual learning of chest radiographic images generalized to untrained images.

Second, our study provided evidence that perceptual learning has both image-specific and general components. Participants trained with 30 images over 24 repetitions were exposed to a more limited set of images. They learned these images better than did participants trained with 60 images over 12 repetitions. This finding showed that training yielded image-specific learning. Nonetheless, when tested with untrained images displayed in an intact format, the two groups of participants performed at comparable levels. Thus, when it comes to the generalizable component, a two-fold increase in repetition traded off with a two-fold decrease in the number of different trained images.

Both expert interviews (van der Gijp et al., 2014) and previous empirical research (Chen et al., 2017) found that greater image variability was more conducive to acquiring transferable skills. In the real world with limited time and resources, an increase in the number of times an image is repeated would necessarily come at the cost of not being able to train on a larger number of different images. Other studies suggest that both mastery of a small set of stimuli and exposure to a diverse number of stimuli contribute to high-level expertise. For example, both the number of games played and the number of study hours in chess correlate with chess rating. However, it is not uncommon for masters to reach similar levels following different amounts of training (Campitelli & Gobet, 2008; Grabner, Stern, & Neubauer, 2007; Howard, 2012). Instead of studying a large number of topics, deliberate practice on specific topics to complete mastery is important for skill acquisition (Campitelli & Gobet, 2008; Charness et al., 2005; Ericsson, 2015). Thus, both stimulus variability and repetition of a limited set of stimuli are likely important.

In perceptual learning of medical images, a previous study found greater improvement with a larger number of different training images (Chen et al., 2017). In contrast, the current study showed that a two-fold increase in repetition largely offset the cost of a two-fold decrease in image variability. Because of the use of just two groups, our study does not answer the question of which combination is optimal. The optimal point of the tradeoff may differ for different types of stimuli.

The third contribution of our study was to clarify the roles of global image processing and local properties in perceptual learning of chest radiographs. Following training, participants were able to classify a trained image as normal or abnormal even when the tumor region was cut out. However, there was no transfer to untrained images. Thus, participants learned incidental properties in the background of a specific image, such as a slightly darker rib cage. These properties did not support generalization, suggesting that in our task, the background regions do not contain signals correlated with tumors.

The small cutout of the tumor region was also a weak signal for the task. Participants improved from pre-test to final post-test. However, the improvement was moderate for both trained and untrained images and was inconsistent across experiments. This finding shows that although local tumor properties were learned from the small region of the tumor, it was not the main signal driving performance.

When the cutout was enlarged to four times the area of the tumor, participants were able to detect tumors in both trained and untrained images. This finding is consistent with Carmody et al. (1981) that radiologists rely on a comparison between tumors and other regions to detect cancer. It further shows that the regions immediately surrounding the tumor were important.

Even though the larger cutout was four times the area of the tumor, it was small compared with the entire image. On average, the area of the larger cutout was only 0.03% of the total image size. Most of the global image properties, such as where the tumor was and what other parts of the image look like, are absent. Yet participants could still detect tumors on the larger cutout, suggesting that they did not rely on image statistics coming from the entire lung. It is likely that by including some of the surrounding areas along with the tumor, the larger cutout provided the key signal for tumor detection - local image contrast between the tumor and its immediate surround. Because we do not have a precise measurement of the size of the tumor relative to the cutout, even the small cutout may have included some surrounding tissues, accounting for why performance was moderate but above chance. Although local contrast but not the larger background was important in our study, it remains possible that more extensive training may lead to greater reliance on the entire image (Chin et al., 2018).

The forced-choice training procedure, with normal and abnormal images presented side by side, does not resemble radiological diagnosis. Nonetheless, our data showed that the training procedure was effective, as demonstrated in the testing sessions that used a more realistic task. The training procedure directed participants’ attention to tumor-specific properties of the images. This is an important component of perceptual learning of complex images. As Kellman (2013) noted, “With practice on a given task, learners come to pick up the relevant information for relevant classifications while ignoring irrelevant variation.” Our use of the two-step procedure with feedback, in which participants first choose the cancerous image and then localize the tumors, guides participants to attend to the relevant region of the images.^3^

**Fig. 7:**
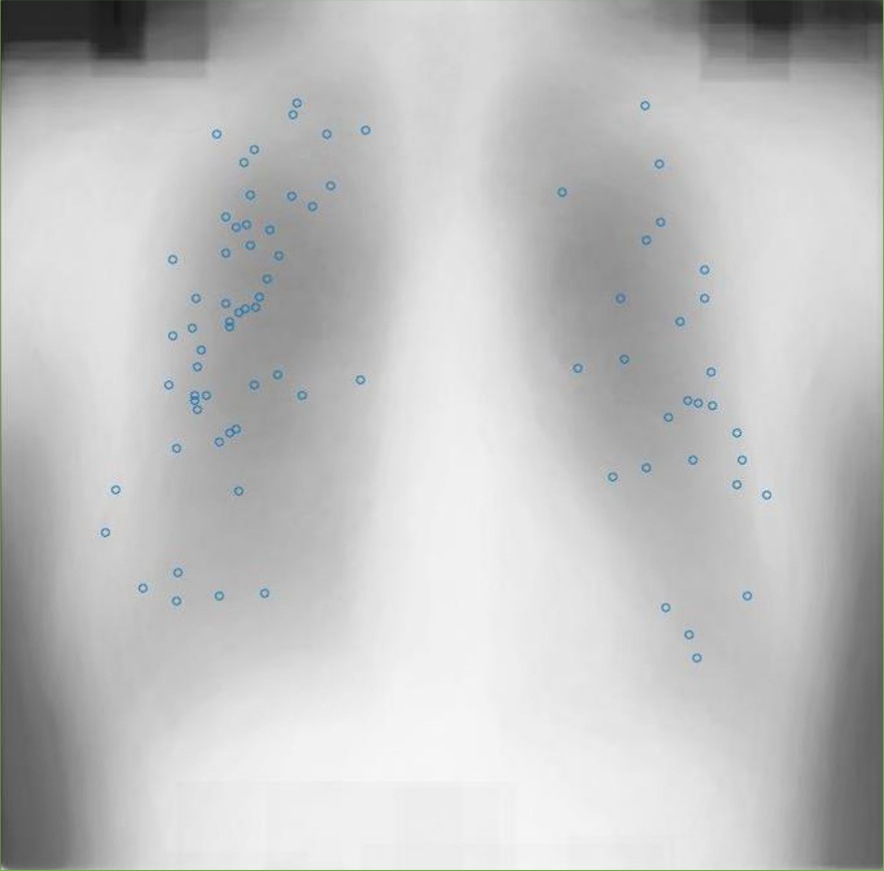
Tumor locations against an average chest radiographic image from the stimuli used in the current study. Each blue dot represents the center location of a tumor from one image

Our study provides initial evidence that participants are able to learn perceptual properties associated with tumor signals. Learning may also have a spatial component, given that the spatial distribution of tumors is constrained by anatomy and etiology. Some cancers tend to cluster in specific locations. For instance, metastases in the brain from primary pelvic or gastrointestinal tumors have a high concentration in the posterior fossa (Delattre, Krol, Thaler, & Posner, 1988). We have previously shown that people are highly sensitive to the location distribution of targets in simulated radiographic images (Sha, Remington, & Jiang, 2018). To examine whether the images used in our study contained such location regularities, we displayed the tumor locations against an “average” chest radiographic image (the average of all the images used in our study). As seen in Fig. 7, the locations span a wide region of the lungs, though the lateral locations appear to contain a greater concentration of tumors. Learning the spatial regularities of the image set may have tuned participants’ spatial attention to the more probable regions. Future studies are needed to further elucidate the role of spatial learning in medical image perception.

## Summary and conclusion

Our study joined a small number of recent findings in demonstrating that perceptual training on complex medical images could yield transferrable perceptual learning. Learning has both a specific component restricted to the trained images and a generalizable component. The background was learned in a largely stimulus-specific manner and evidenced little transfer to novel images. The transfer was observed if the entire image was presented, or if a small region about four times the size of the tumor was displayed. The contrast between the tumor region and surrounding areas may contain the key properties learned in our study. Whether the same conclusions hold with other types of radiological images, such as mammography or ultrasound images, remains to be tested in the future.

This study constituted a proof of concept that even the assessment of complex radiological images could benefit from perceptual learning. The finding may have practical implications, such as in the training of medical students. Such training would benefit from additional techniques, such as the adaptive response-time-based sequencing (ARTS) system introduced by Kellman and colleagues. In an earlier study, they successfully implemented ARTS in training first-year and second-year medical students in identifying skin lesions (Kellman, 2013; Kellman & Garrigan, 2009). Our study also raised the question of how to induce perceptual learning of the most subtle tumor images, as participants remained unable to detect tumors on untrained images with a subtlety level of 1. Computerized perceptual learning, like the type used here, may facilitate the initial acquisition of perceptual skills. Nonetheless, prolonged training, along with conceptual knowledge of pathology and imaging techniques, will be necessary to achieve high levels of expertise.

## Data Availability

Consent forms used in this study contain a clause of data privacy that prevents the release of raw data. However, de-identified, aggregated data are available upon reasonable request.

## Appendix

**Table 5:** Response criterion in the pre-test and final post-test sessions

Image format	Training status	Pre-test	Final post-test	Statistical results (ANOVA)
Intact (*N* = 60)	Untrained	−0.29	−0.17	No change in criterion. No main effects or interaction, *p*s > .24.
	Trained	−0.24	−0.13	
Small cutout (*N* = 60)	Untrained	0.65	0.25	An initial bias toward “normal” was reduced (*p* < .002). No other effects (*ps* > .16*)*
	Trained	0.69	0.35	
Large cutout (*N* = 12)	Untrained	0.30	0.28	No change in criterion. No main effects or interaction, *p*s > .25
	Trained	0.40	0.19	
Background (*N* = 48)	Untrained	0.09	0.09	No change for untrained images. Response became more liberal for trained images (*p* < .04) Significant interaction *p* < .001
	Trained	0.16	−0.09	

Positive values indicate a bias in reporting “normal”. Negative values indicate a bias in reporting “abnormal”. *ANOVA* analysis of variance

As seen in Table 5 in Appendix, changes in response criterion across pre-test to the final post-test varied by the format of the testing image. Intact images were associated with a slight bias toward responding “abnormal”, and this did not change over time. Small cutouts were associated with an initial bias toward responding “normal”, and this bias was attenuated after training. Large cutouts were associated with a slight bias toward reporting “normal”, and this did not change over time. Background regions were associated with a small bias toward reporting “normal” before training. After training, this bias remained when classifying untrained images, but changed to a slight bias toward reporting “abnormal” when classifying trained images.

## Footnotes

1 Sample sizes were in the range of 4–8 in the studies of Censor and Sagi (2009), Dosher, Han and Lu (2010), and Yu, Klein, and Levi (2004) on perceptual learning of texture, orientation, or contrast discrimination. The sample size in a study that trained participants on more complex stimuli (“greebles”) was 12 (Gauthier, Williams, Tarr, & Tanaka, 1998).

2 The image size differed between training and testing because the tasks involved a different number of images per display. The training task presented two images side by side, limiting the size of each image that could be displayed. The classification task used in testing presented a single image in the center, allowing for a larger presentation. Images also differed in location - lateralized in training and at the center in testing.

3 In pilot data we found that providing accurate feedback about tumor location was essential for learning. In 17 participants tested in a pilot experiment similar to the training in our experiment 3, the computer provided correct feedback on which image contained tumor, but marked a wrong tumor location on the cancerous image. These participants failed to improve - their classification of which image was normal and which cancerous remained flat across 360 training trials.
